# Interplay Between the MicroRNA miR-152 and Quercetin in the Control of Ovarian Granulosa Cell Functions

**DOI:** 10.1007/s43032-024-01728-z

**Published:** 2024-10-30

**Authors:** Alexander V. Sirotkin, Zuzana Fabová, Zuzana Kislíková, Barbora Loncová, Miroslav Bauer, Maria Bauerová, Abdel Halim Harrath

**Affiliations:** 1 Faculty of Natural Sciences, Constantine the Philosopher University in Nitra, Nitra, Slovakia; 2 Research Institute for Animal Production Nitra, National Agricultural and Food Center (NPPC), Hlohovecká 2, Lužianky, 951 41 Slovakia; 3 Department of Zoology, College of Science, King Saud University, Riyadh, 11451 Saudi Arabia; 4 Department of Zoology and Anthropology, Constantine the Philosopher University, Tr. A. Hlinku 1, 949 74, Nitra, Slovakia

**Keywords:** miR-152, Quercetin, Ovary, Hormones, Apoptosis, Proliferation

## Abstract

In the present study, we examined the functional interrelationships between microRNAs and plant polyphenols in the regulation of ovarian cell functions. For this purpose, we compared the basic functions of porcine ovarian granulosa cells with or without transfection with miR-152 mimics that were cultured with or without quercetin. The expression levels of miR-152, cell viability, cell proliferation (accumulation of proliferating cell nuclear antigen, PCNA), apoptosis (accumulation of Bax) and the release of progesterone, estradiol, and insulin-like growth factor I (IGF-I) were analyzed by real-time quantitative polymerase chain reaction (RT‒qPCR), the Trypan blue exclusion test, quantitative immunocytochemistry, and enzyme-linked immunosorbent assays (ELISAs). Transfection of cells with miR-152 mimics increased miR-152 expression, reduced cell viability, proliferation, apoptosis, and estradiol output, and promoted the release of progesterone and IGF-I. Quercetin decreased all measured parameters. Moreover, quercetin promoted the effect of miR-152 on cell viability, apoptosis, and estradiol and mitigated the effect of miR-152 on cell proliferation and IGF-I output. For instance, miR-152 mimics promoted the effect of quercetin on cell viability, apoptosis, and estradiol but prevented the effect of quercetin on PCNA. These observations demonstrated the involvement of miR-152 and quercetin in the control of ovarian cell functions and their functional interrelationships, mainly synergism, in the regulation of these functions.

## Introduction

The application of plant molecules as safe regulators of physiological processes and as medicines remains popular. Several medicinal plants and plant polyphenols [1], including the flavonoid quercetin [2], can be used to prevent and treat pathological conditions of the female reproductive system, including infertility, cancer, and polycystic ovarian syndrome. For example, quercetin has been shown to directly inhibit porcine ovarian granulosa cell function [3–6]. Quercetin can prevent the stimulatory effect of follicle stimulating hormone (FSH) [2, 5] with other physiological regulators of ovarian functions, for example with non-coding RNAs (see below) have not yet been studied.

The use of alternatives and additives to classical phototherapeutic and pharmacological methods for the control and treatment of conditions affecting female reproductive processes may have effects through RNA interference [7, 8]. Epigenetic approaches include the application of small noncoding RNAs that affect the translation and degradation of specific coding RNAs to control, detect, and treat female reproductive disorders [9–11]. One such noncoding RNA, the microRNA (miRNA or miR) miR-152, was shown to be a potent regulator of the basic functions of cultured healthy porcine granulosa cells [12] and an inhibitor of the basic functions of healthy [13, 14] and cancerous [15, 16] human ovarian cells.

Despite the widespread applicability of both phytotherapeutic and epigenetic approaches in reproductive biology and medicine, the functional interrelationships between biologically active plant molecules and small RNAs in the control of ovarian cell function have been poorly investigated. Plant polyphenols, including quercetin, genistein, curcumin, and resveratrol, can affect the expression of several miRNAs in non-ovarian cells [2] and in the ovaries [17]. These findings indicate that these polyphenols can affect ovarian cancer cells via changes in the miRNAs that regulate proliferation and apoptosis [17, 18]. In contrast, effects of miRNAs on polyphenols in the ovary are also possible. MiR-152 not only affects the basic functions of cultured healthy porcine granulosa cells but also prevents the influence of the plant flavonoid apigenin on some cell functions. On the other hand, apigenin was shown to affect miR-152 [12]. It remains to be established whether miRNAs can interact with other plant polyphenols, for example of quercetin, to control ovarian cell functions.

We examined whether quercetin can affect miR-152 activity on ovarian cells and vice versa and whether miR-152 can affect the response of ovarian cells to quercetin. For this purpose, we compared the basic functions of porcine ovarian granulosa cells with or without transfection with miR-152 mimics and cultured them with or without quercetin. The expression of miR-152, cell viability, cell proliferation (accumulation of proliferating cell nuclear antigen, PCNA), and apoptosis (accumulation of Bax) and the release of progesterone, estradiol and insulin-like growth factor I (IGF-I) were analyzed.

## Materials and Methods

### Oligonucleotides

MiR-152 mimics (double-stranded RNAs that mimic mature endogenous miR-34a and increase miRNA activity, representing a gain-of-function assay) and their appropriate negative controls (NCs) labeled with fluorescein (Table 1) were purchased from GenePharma Co., Ltd. (Shanghai, China). These oligonucleotides were synthesized and purified by using high-performance liquid chromatography (according to the manufacturer’s data, more than 97% purity was detected by using mass spectrometry).

**Table 1:** The sequences of miRNA mimics

Oligonucleotides	Sequence	
miR-152 mimics	Sense	5′-UCAGUGCAUGACAGAACUUGG-3′
	Antisense	5′-AGUUCUGUCAUGCACUGAUU-3′
miR-NC (negative control) mimics	Sense	5′-UUCUCCGAACGUGUCACGUTT-3′
	Antisense	5′-ACGUGACACGUUCGGAGAATT-3′

### Preparation, Transfection, Culture, and Processing of Ovarian Granulosa Cells

The ovarian cells were isolated, cultured, transfected and analyzed as it was described previously [3, 5, 6, 12]. In the present experiments, the ovaries were collected from prepubertal Landrace gilts (6–8 months of age) at the slaughterhouse of Chovmat F.U. in Rastislavice (Slovakia). The ovaries were individually stored in a thermos with a physiological solution at room temperature and processed within 6 h of slaughter.

Ovarian granulosa cells were isolated from porcine ovarian (4.5–6.5 mm diameter) follicles without visible signs of atresia (including weak vascularization, thin follicular walls, and pale follicular fluid) by using aspiration with a syringe. After aspiration and cell isolation *via* centrifugation for 10 min at 1,500 rpm, the granulosa cells were washed in sterile Dulbecco’s Modified Eagle Medium/Nutrient Mixture F-12 (DMEM/F12) 1:1 medium (BioWhittakerTM; Lonza, Verviers, Belgium) and resuspended in the same medium supplemented with 10% fetal calf serum (BioWhittakerTM) and 1% antibiotic-antimycotic solution (Sigma‒Aldrich, St. Louis, MO, USA). Cells were counted by using a Bürker chamber, and their concentration was adjusted based on the required volume (10^6^ cells/ml medium). The cell suspension was dispensed in 24-well culture plates (NuncTM, Roskilde, Denmark; 1 ml suspension/well) for enzyme-linked immunosorbent assays (ELISA) or onto 16-well chamber slides (Nunc, Inc., International, Naperville, IL, USA; 200 µl/well) for immunocytochemistry. The cells were precultured in medium at 37.5 °C in 5% CO_2_ until a 75% confluent monolayer was formed (2–3 days).

The experimental cells were transfected with miR-152 mimics and their negative controls (NCs) by using Lipofectamine^®^ RNAiMAX Transfection Reagent (Invitrogen, Carlsbad, CA, USA) according to the manufacturer’s protocol. For each transfection, a final oligonucleotide concentration of 25 nM was used. The control groups consisted of nontransfected cells or cells transfected with NC. After transfection for 24 h, the granulosa cells were cultured with or without quercetin (0, 1, 10, or 100 µg/ml; AppliChem GmbH, Darmstadt, Germany). These doses were comparable to the effective doses of quercetin used in previous similar in vitro experiments [2, 6]. Just before its addition to the cells, plant isoflavone was dissolved first in 0.1% dimethyl sulfoxide (DMSO) and then in culture medium. The control groups consisted of nontransfected or transfected cells with no quercetin treatment.

After culture, the culture medium and cells were processed for immunocytochemistry, reverse transcription-quantitative PCR (RT‒qPCR), and ELISAs.

### Cell Viability Test

Cell viability was determined by using the Trypan blue exclusion test (0.4%), as described previously [19, 20]. Briefly, the medium was removed from the culture plates after the granulosa cells were incubated. Subsequently, the cell monolayer was subjected to Trypan blue staining (Sigma‒Aldrich) for 15 min. After Trypan blue treatment, the cells were fixed for 30 min in 4% paraformaldehyde. After fixation, the plates were washed with a physiological solution and subjected to microscopic inspection (magnification: 400×). The ratio of dead (stained) cells to the total cell count was calculated.

### Immunocytochemical Analysis of Proliferation and Apoptosis Markers

The markers of proliferation (PCNA) and apoptosis (Bax) were detected *via* immunocytochemistry by using primary mouse monoclonal antibodies against either PCNA or caspase 3 (diluted 1:500 in phosphate buffered saline (PBS); Santa Cruz Biotechnology, Inc., Dallas, TX, USA), secondary swine antibody against mouse IgG (diluted 1:1,000; Santa Cruz Biotechnology, Inc.) labeled with horseradish peroxidase (Servac, Prague, Czech Republic), or secondary goat antibody against mouse IgG (Sigma‒Aldrich) labeled with CruzFluor™ 594 (CFL 594, diluted 1:500). Horseradish peroxidase-labeled cells were stained with 3,3’-diaminobenzidine (DAB) substrate (Roche Diagnostics GmbH, Mannheim, Germany). Moreover, cells labeled with CFL 594 were mounted in VECTASHIELD Antifade Mounting Medium with 4′,6-diamidino-2-phenylindole (DAPI), which is a selective stain for cell nuclear DNA (Vector Laboratories, Inc., Burlingame, CA, USA). DAPI- and CFL 594-labeled secondary antibodies were detected by fluorescence microscopy. Cells treated without the primary antibody were used as negative controls. In addition, the number of stained cells and the location of intracellular molecules were determined based on the brown coloration of DAB peroxidase or the red fluorescence emitted by the CFL 594 label by using a light or fluorescence microscope (Leica Microsystems, Wetzlar, Germany) and IM500 Leica software. The ratio of stained cells to the total number of cells was determined.

### RT‒qPCR for miR-152

After treatment with miR-152 mimics for 48 h, total RNA from transfected granulosa cells was extracted by using TRIzol reagent (Invitrogen) according to the manufacturer’s instructions. The concentration and quality of total RNA were measured by using a UV spectrophotometer (Bio-Rad, Inc.; Hercules, CA, USA). The expression levels of mature miR-152 were quantified by using the Hairpin-it-miRNAs qPCR kit (GenePharma Co., Ltd.) on an Applied Biosystems™ 7500 Fast Instrument (Thermo Fisher Scientific). The amplification thermocycling conditions were as follows: initial denaturation at 95 °C for 3 min, followed by 40 cycles at 95 °C for 10 s, annealing and elongation at 60 °C for 10 s, and 60 °C for 60 s. U6 small nuclear RNA (snRNA) was used as an internal control, and relative gene expression was calculated by using the 2^–ΔΔCt^ method [21]. The sequences of the utilized primers (Table 2) were designed and synthesized by GenePharma Co., Ltd. All of the samples were analyzed in triplicate from the same RNA preparation, and the mean values were calculated.

**Table 2:** The sequences of gene primers for RT-qPCR

Gene symbol	Primer sequence	
miR-152	Forward	5′-TGGTTCGCTCAGTGCATGA-3′
	Reverse	5′-TATGGTTGTTCACGACTCCTTCAC-3′
U6	Forward	5′-CTCGCTTCGGCAGCACA-3′
	Reverse	5′-AACGCT TCACGAATTTGCGT-3′

### Enzyme-Linked Immunosorbent Assay (ELISA)

The concentrations of progesterone, *17β-*estradiol, and IGF-1 were determined in 25 µl aliquots of the incubation medium by using ELISAs according to the manufacturer’s instructions (LDN Immunoassays and Services, Nodhorn, Germany). The characteristics of these assays are presented in Table 3. This ELISA was validated for culture medium samples by using dilution tests.

**Table 3:** Characteristics of the immunoassays used in experiments

Substance assayed	Specificity of assay (cross-reactivity of antiserum)	Sensitivity of assay (ng/ml)	Coefficient of variation (%)	
			Intra-assay	Inter-assay
**Progesterone**	≤ 1.1% with 11-desoxycorticosterone, ≤ 0.35% with pregnenolone, ≤ 0.30% 17α-OH with progesterone, ≤ 0.20% with corticosterone, ˂0.10% with estriol, 17β-estradiol, testosterone, cortisone and 11-desoxycortisol, ˂0.02% with DHEA-S and cortisol	0.045	5.4	5.59
***17β*** **-estradiol**	≤ 9.5% with fulvestrant, ≤ 4.2% with estrone, ≤ 3.8% with E2-3-glucuronide, ≤ 3.6% with E2-3-sulphate, ≤ 0.4% with estriol, ˂0.1% with androstenedione, 17-hydroxyprogesterone, corticosterone, pregnenolone, E2-17-glucuronide, progesterone, and testosterone	0.0062	6.4	4.5
**IGF-I**	100% with IGF-I, ≤ 3.3% with insulin, and 1.02% with IGF-II	9.75	7.39	12.63

### Statistical Analysis

The data from this study are reported as the means of values that were obtained in three separate experiments performed on separate days with different groups of granulosa cells, each obtained from at least six ovaries. Each experimental group was represented by four culture wells containing ovarian granulosa cells. For the immunocytochemical analysis, the proportion of cells containing antigens was calculated for at least 1,000 cells per well. For the ELISAs, blank control values were subtracted from corresponding values that were determined for media-containing cells to exclude any nonspecific background (less than 10% of the total values). The rates of hormone secretion were calculated per 10^6^ viable cells/day. Significant differences between the groups were determined by using the Shapiro‒Wilk normality test and Student’s t test, as well as one-way ANOVA followed by Tukey’s test or the Newman-Keul post hoc test with SigmaPlot 11.0 (Systat Software, GmbH, Erkrath, Germany). Differences were considered statistically significant at p levels less than 0.05 (*p* < 0.05).

## Results

The ovarian granulosa cells collected after culture were viable, expressed markers of proliferation (PCNA) and apoptosis (Bax), and released substantial amounts of steroid hormones and IGF-I. The measured parameters were affected by transfection with miR-152 and quercetin alone or in combination.

### Efficiency of Transfection

**Fig. 1:**
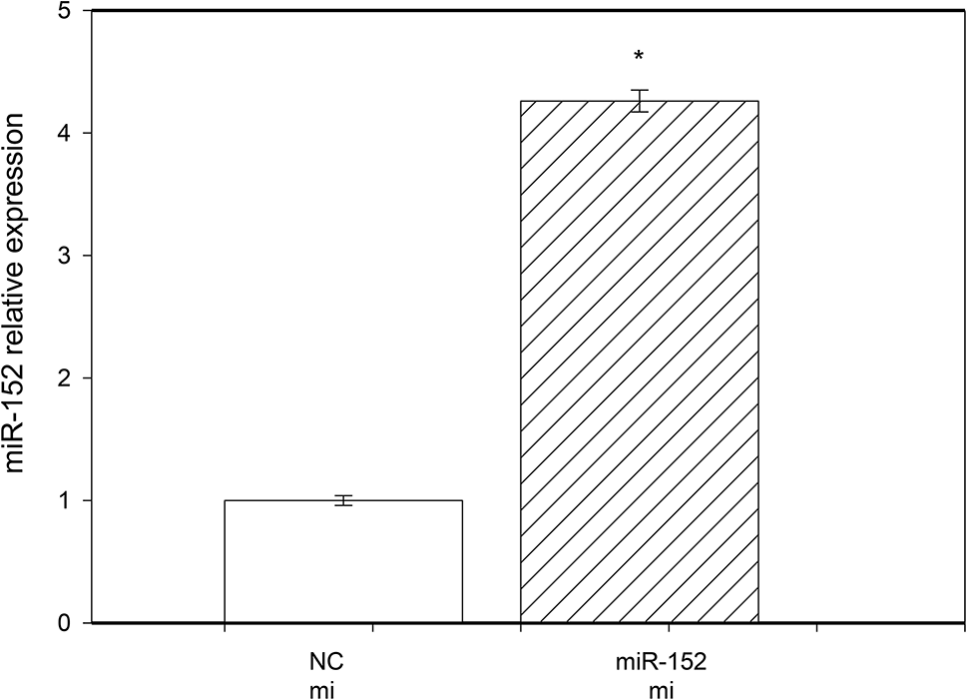
Evaluation of the miR-152 expression level by reverse transcription-quantitative polymerase chain reaction (RT‒qPCR) in cells transfected with miR-152 mimics (mi) or with the negative control oligonucleotide (NC). The values are the means ± SEMs. Significance vs. the control: *, *P* < 0.05

RT‒qPCR revealed that the expression of miR‒152 in the ovarian granulosa cells transfected with miR‒152 mimics was several times greater than that in the cells transfected with the negative control (NC) oligonucleotide (Fig. 1).

### The Effects of miR-152 Mimics Alone

**Fig. 2:**
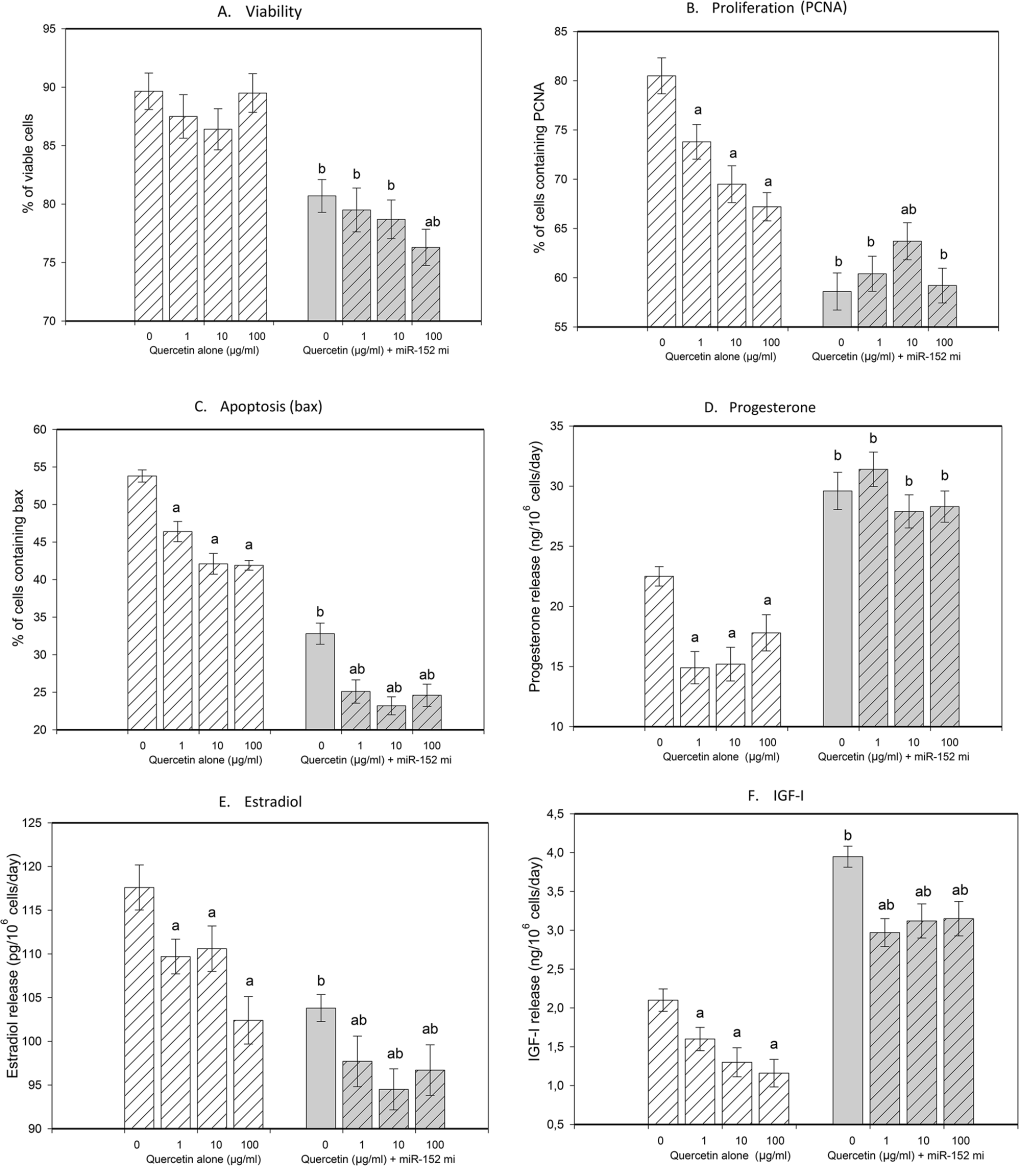
Effect of the administration of quercetin (0, 1, 10, 100 µg/ml) alone or in combination with miR-152 mimics (mi) on cell viability (**A**); the accumulation of PCNA (**B**) and Bax (**C**); and the release of progesterone (**D**), estradiol (**E**), and IGF-I (**F**) in cultured porcine ovarian granulosa cells. The results show (**a**) the effect of quercetin: significant (*P* < 0.05) differences between cells treated without (0 µg/ml) and with quercetin; (**b**) the effect of miR-152 mimics: significant (*P* < 0.05) differences between the corresponding groups of cells with and without transfection with miR-152 mimics. The results are expressed as the mean ± SEM from at least 3 independent experiments

Transfection of cultured porcine granulosa cells with miR-152 mimics significantly reduced cell viability (Fig. 2A), the proportion of cells containing the proliferation-related peptide PCNA (Fig. 2B) and the percentage of Bax-positive cells (Fig. 2C) (see the miR-152mi group, quercetin at a dose of 0 µg/ml). Furthermore, transfection with miR-152 mimics increased progesterone release (Fig. 2D), decreased estradiol output (Fig. 2E) and increased IGF-I release (Fig. 2F).

### The Effects of Quercetin Alone

Quercetin, when added alone (see cells not transfected with miR-152 mimics), decreased the viability of cultured ovarian granulosa cells (Fig. 2A), the accumulation of PCNA (Fig. 2B) and apoptosis (Fig. 2C) at all doses tested. Furthermore, the addition of quercetin reduced the release of progesterone (Fig. 2D), estradiol (Fig. 2E) and IGF-I (Fig. 2F) at all the tested doses.

### The Ability of Quercetin to Modify the Effect of miR-152 Mimics

The addition of quercetin promoted the inhibitory effect of miR-152 on cell viability (Fig. 2A) but not the accumulation of PCNA (Fig. 2B): quercetin at doses of 1–100 µg/ml did not affect PCNA accumulation in transfected cells, and in the presence of 10 µg/ml quercetin, PCNA accumulation in transfected cells was even greater than that in the absence of quercetin (at a dose of 0 µg/ml). Quercetin promoted the inhibitory effect of miR-152 on the accumulation of Bax (Fig. 2C), but this treatment did not influence the stimulatory effect of miR-152 on progesterone output (Fig. 2D). The addition of quercetin promoted the inhibitory effect of miR-152 on the release of estradiol (Fig. 2E), but it reduced the stimulatory effect of miR-152 on IGF-I release (Fig. 2F).

### The Ability of miR-152 Mimics to Modify the Effect of Quercetin

Transfection of cells with miR-152 mimics promoted the inhibitory effect of quercetin on granulosa cell viability (Fig. 2A), the percentage of Bax-positive cells (Fig. 2C), and the release of estradiol (Fig. 2E) and IGF-I (Fig. 2F), but it changed the inhibitory effect of quercetin on PCNA accumulation from inhibitory to stimulatory (Fig. 2B). Furthermore, in cells transfected with miR-152 mimics, quercetin failed to affect progesterone release (Fig. 2D).

## Discussion

The high viability of cultured granulosa cells, the high expression of their proliferation marker PCNA, the secretion of substantial amounts of steroid hormones and IGF-I by these cells, and the cell response to the analyzed treatments suggested that the cell cultures used were viable, active, and useful as a model for investigating the regulators of their functions. Moreover, the high overexpression of miR-152 in cells after transfection with miR-152 mimics suggested that this transfection was successful.

### The Effects of miR-152 Mimics Alone

In the present study, transfection with miR-152 mimics decreased cell viability, proliferation, apoptosis, and estradiol release. These observations are consistent with previous evidence showing the involvement of miR-152 in the control of healthy [13, 14] and cancerous [15, 16] ovarian cells from human and porcine ovarian granulosa cells [12]. Some differences between the present and previous observations regarding the influence of miR-152 on particular ovarian characteristics could be explained by the variability in the initial state of the ovarian cells used in the experiments. Nevertheless, the present observations suggest that miR-152 can decrease ovarian cell function. The hierarchical functional interrelationships between these functions should not be excluded. For example, estradiol is considered a promoter of ovarian cell proliferation, viability, and follicular health and development [22]. Therefore, miR-152 may suppress ovarian cell proliferation by decreasing estradiol production. The suppressive effect of miR-152 on these processes observed in the present study supports previous evidence indicating the usefulness of this miRNA for the suppression of healthy [13, 14] and cancerous [15, 16] human ovarian cells.

In our experiments, miR-152 overexpression was associated with a decrease in ovarian cell apoptosis and an increase in progesterone and IGF-I release. These observations suggest that miR-152 can promote ovarian cell functions such as ovarian cell luteinization, which is associated with an increase in progesterone and a decrease in estradiol production [22]. The antiapoptotic effect of miR-152 may be due to the increase in progesterone and IGF-I production, the antiapoptotic effects of which are well documented [22]. It is unlikely that these hormones could be endocrine mediators of the suppressive effect of miR-152 on ovarian cell proliferation and viability because both progesterone and IGF-I are considered promoters of cell proliferation and viability [22, 23].

Taken together, the present observations confirm the previous evidence for the involvement and potential applicability of miR-152 in the control of ovarian functions, although the mechanisms and physiological role of this miRNA require further study.

### The Effects of Quercetin Alone

In the present study, the addition of quercetin resulted in a decrease in all the measured ovarian cell parameters. These observations are consistent with the previous reports demonstrating the suppressive effect of this plant flavonoid on female reproductive processes including functions of cultured porcine ovarian granulosa cells [2–6].

The role of quercetin as an endocrine mediator of some ovarian cell functions may be proposed because in the present and previous studies, quercetin suppressed steroid hormones and IGF-I, which are known promoters of cell proliferation and viability. Nevertheless, understanding the hierarchical interrelationships between quercetin-dependent events and the mechanisms of quercetin action requires further study.

The suppressive effect of quercetin on ovarian cell function indicates the potential hazard of the consumption of quercetin-containing plants for female reproductive processes. However, this finding supports previous evidence [2] for the applicability of quercetin for the prevention and treatment of ovarian cancer, which is associated with increased viability and proliferation of cells.

Therefore, the present observations demonstrate the suppressive influence of quercetin on basic ovarian cell functions, which could be attributed to the application of this plant polyphenol.

### Interplay between Quercetin and miR-152

The present study first identifies the ability of quercetin to modify the effect of miRNAs on ovarian cells by promoting the influence of miR-152 on cell viability, apoptosis, and the release of estradiol and to mitigate and even reverse the effects of miR-152 on ovarian cell proliferation and IGF-I release. In contrast, the present study first demonstrated the ability of miR-152 to change the response of ovarian cells to quercetin administration: miR-152 promoted the effects of quercetin on cell viability, apoptosis, and the release of estradiol and IGF-I. These findings, together with the similarity of the effects of miR-152 and quercetin on ovarian cells, suggest synergistic and probably similar mechanisms of action of this miR and plant polyphenol. Such a common mechanism may involve estradiol levels. Quercetin is a phytoestrogen that can affect steroid hormone receptors and, therefore, alter the reception and secretion of steroid hormones. The influence of quercetin on ovarian steroidogenesis has been demonstrated previously [2] and in the present study. The ability of miR-152 to affect steroidogenesis has been shown previously [12, 13] and in the present study. Steroid hormones are known regulators of ovarian cell function [24]. Therefore, the first site of cooperation between quercetin and miR-152 in the control of ovarian cell functions may be estradiol.

However, some evidence indicates not only synergistic but also antagonistic relationships between quercetin and miR-152. In the present study, quercetin and miR-152 had opposite effects on IGF-I release, while miR-152 prevented and reversed the effect of quercetin on ovarian cell proliferation. These observations indicate that IGF-I could be the next site of crosstalk between these regulators. Such crosstalk could define the final effects of polyphenols and miRs on cell proliferation, which is under the stimulatory control of IGF-I [24].

The ability of quercetin to modify miR-152 activity indicates the next mechanism of action of this polyphenol, which has not been reported previously and can affect not only basic ovarian cell functions but also the effects of their epigenetic regulator, miR. This effect of quercetin should be considered when consuming quercetin-containing plants during the application of miR for medical purposes. Furthermore, the synergistic and mutual effects of quercetin and miR-152 on ovarian cells may be useful for improving ovarian cancer treatment via the use of miR-152 [25] and quercetin [2]. The combination of quercetin and miR-152 increases the therapeutic effect of each molecule. However, this hypothesis requires validation through in vivo studies.

Taken together, the present observations demonstrate the ability of quercetin to modify the influence of miR-152 on ovarian cells and the ability of miR-152 to promote the influence of quercetin on these cells. This study helps elucidate the interrelationships between different physiological regulators of female reproduction and outline possible approaches to increase the efficacy of their application.

Although the mechanisms of action, the crosstalk of quercetin and miR-152 in the control of female reproductive processes, and their applications require further study, the present observations (1) confirm the involvement of both miR-152 and quercetin in the control of basic ovarian cell functions and (2) demonstrate for the first time the functional interrelationships, mainly the synergism of these regulators, in the control of these functions. These observations indicate that this interrelationship is mediated by estradiol and IGF-I.

## Data Availability

The primary data can be provided upon request.

## References

[1] Sirotkin AV, Kolesarova A. Environmental contaminants and medicinal plants action on female reproduction. Academic; 2022.

[2] Sirotkin AV. Quercetin action on health and female reproduction in mammals. Crit Rev Food Sci Nutr. 2023. Epub ahead of print. 10.1080/10408398.2023.2256001. 10.1080/10408398.2023.2256001 37698182

[3] Tarko A, Štochmal’ová A, Jedličková K, Hrabovszká S, Vachanová A, Harrath A, Alwasel S, Alrezaki A, Kotwica J, Baláži A. Effects of benzene, quercetin, and their combination on porcine ovarian cell proliferation, apoptosis, and hormone release. Arch Anim Breed. 2019;62:345–51. 31807645 10.5194/aab-62-345-2019 PMC6852862

[4] Tarko A, Štochmaľová A, Harrath A, Kotwica J, Baláži A, Sirotkin A. Quercetin can affect porcine ovarian cell functions and to mitigate some of the effects of the environmental contaminant toluene. Res Vet Sci. 2023;154:89–96. 36516587 10.1016/j.rvsc.2022.12.005

[5] Sirotkin AV, Hrabovszká S, Štochmaľová A, Grossmann R, Alwasel S, Harrath AH. Effect of quercetin on ovarian cells of pigs and cattle. Anim Reprod Sci. 2019;205:44–51. 30981564 10.1016/j.anireprosci.2019.04.002

[6] Sirotkin AV, Štochmaľová A, Alexa R, Kadasi A, Bauer M, Grossmann R, Alrezaki A, Alwasel S, Harrath AH. Quercetin directly inhibits basal ovarian cell functions and their response to the stimulatory action of FSH. Eur J Pharmacol. 2019;860:172560. 31344364 10.1016/j.ejphar.2019.172560

[7] Nemeth K, Bayraktar R, Ferracin M, Calin GA. Non-coding RNAs in disease: from mechanisms to therapeutics. Nat Rev Genet. 2024;25(3):211–32. 10.1038/s41576-023-00662-1. 10.1038/s41576-023-00662-1 37968332

[8] Ranasinghe P, Addison ML, Dear JW, Webb DJ. Small interfering RNA: Discovery, pharmacology and clinical development—An introductory review. Br J Pharmacol. 2023;180(21):2697–720. 36250252 10.1111/bph.15972

[9] Sirotkin AV. Application of RNA interference for the control of female reproductive functions. Curr Pharm Design. 2012;18(3):325–36. 10.2174/138161212799040376 22229568

[10] Nouri N, Shareghi-Oskoue O, Aghebati-Maleki L, Danaii S, Ahmadian Heris J, Soltani-Zangbar MS, Kamrani A, Yousefi M. Role of miRNAs interference on ovarian functions and premature ovarian failure. Cell Communication Signal. 2022;20(1):1. 10.1186/s12964-022-00992-3 PMC9783981 36564840

[11] Rehman U, Parveen N, Sheikh A, Abourehab MAS, Sahebkar A, Kesharwani P. Polymeric nanoparticles-siRNA as an emerging nano-polyplexes against ovarian cancer. Colloids Surf B Biointerfaces. 2022;218:112766. 10.1016/j.colsurfb.2022.112766. 10.1016/j.colsurfb.2022.112766 35994990

[12] Fabová Z, Kislíková Z, Loncová B, Bauer M, Harrath A, Sirotkin A. MicroRNA miR-152 can support ovarian granulosa cell functions and modify apigenin actions. Domest Anim Endocrinol. 2023;84:106805. 37354873 10.1016/j.domaniend.2023.106805

[13] Sirotkin AV, Ovcharenko D, Grossmann R, Lauková M, Mlynček M. Identification of MicroRNAs controlling human ovarian cell steroidogenesis via a genome-scale screen. J Cell Physiol. 2009;219(2):415–20. 19194990 10.1002/jcp.21689

[14] Sirotkin AV, Lauková M, Ovcharenko D, Brenaut P, Mlynček M. Identification of microRNAs controlling human ovarian cell proliferation and apoptosis. J Cell Physiol. 2010;223(1):49–56. 20039279 10.1002/jcp.21999

[15] Qin W, Xie W, He Q, Sun T, Meng C, Yang K, Luo Y, Yang D. MicroRNA-152 inhibits ovarian cancer cell proliferation and migration and may infer improved outcomes in ovarian cancer through targeting FOXP1. Experimental Therapeutic Med. 2018;15(2):1672–9. 10.3892/etm.2017.5529 PMC5774449 29434752

[16] Liu W, Zhang L, Wang J, Wang X, Sun H. Analysis of the inhibitory effects of miR–124 and miR-152 on human epithelial ovarian cancer xenografts in a nude mouse model. Oncol Lett. 2019;17(1):348–54. 30655773 10.3892/ol.2018.9612 PMC6313158

[17] Badehnoosh B, Rajabpoor Nikoo N, Asemi R, Shafabakhsh R, Asemi Z. MiRNAs: emerging agents for Therapeutic effects of Polyphenols on Ovarian Cancer. Mini Rev Med Chem. 2024;24(4):440–52. 37587814 10.2174/1389557523666230816090138

[18] Khan K, Javed Z, Sadia H, Sharifi-Rad J, Cho WC, Luparello C. Quercetin and MicroRNA interplay in apoptosis regulation in ovarian cancer. Curr Pharm Design. 2021;27(20):2328–36. 10.2174/1381612826666201019102207 33076802

[19] Perry SW, Epstein LG, Gelbard HA. In situ trypan blue staining of monolayer cell cultures for permanent fixation and mounting. Biotechniques. 1997;22(6):1020–4. 9187742 10.2144/97226bm01

[20] Uzuner SÇ. Development of a direct trypan blue exclusion method to detect cell viability of adherent cells into ELISA plates. Celal Bayar Univ J Sci. 2018;14(1):99–104.

[21] Livak KJ, Schmittgen TD. Analysis of relative gene expression data using real-time quantitative PCR and the 2– ∆∆CT method. Methods. 2001;25(4):402–8. 11846609 10.1006/meth.2001.1262

[22] Sirotkin AV, Kisova G, Brenaut P, Ovcharenko D, Grossmann R, Mlyncek M. Involvement of microRNA Mir15a in control of human ovarian granulosa cell proliferation, apoptosis, steroidogenesis, and response to FSH. Microrna. 2014;3(1):29–36. 25069510 10.2174/2211536603666140227232824

[23] Jozkowiak M, Hutchings G, Jankowski M, Kulcenty K, Mozdziak P, Kempisty B, Spaczynski RZ, Piotrowska-Kempisty H. The stemness of human ovarian granulosa cells and the role of resveratrol in the differentiation of MSCs—a review based on cellular and molecular knowledge. Cells. 2020;9(6):1418. 32517362 10.3390/cells9061418 PMC7349183

[24] Sirotkin AV. Regulators of ovarian functions. New York: Nova Science; 2014.

[25] Friedrich M, Pracht K, Mashreghi MF, Jäck HM, Radbruch A, Seliger B. The role of the miR-148/‐152 family in physiology and disease. Eur J Immunol. 2017;47(12):2026–38. 28880997 10.1002/eji.201747132

